# Inequalities in oral health among adolescents in Gangneung, South Korea

**DOI:** 10.1186/s12903-018-0533-3

**Published:** 2018-04-24

**Authors:** Se-Hwan Jung, Myoung-Hee Kim, Jae-In Ryu

**Affiliations:** 1 Department of Preventive Dentistry, College of Dentistry, Gangneung-Wonju University, 120 Gangneungdaehag-ro, Gangneung City, Gangwon Province 25457 South Korea; 2 Center for Health Equity Research, People’s Health Institute, 36 Sadangro 13-gil, 2nd floor, Dongjak-gu, Seoul, 07004 South Korea; 3 Department of Preventive and Social Dentistry, College of Dentistry, Kyung Hee University, 26 Kyungheedae-ro, Dongdaemun-gu, Seoul, 02447 South Korea

**Keywords:** Adolescent health, Schools, Socioeconomic factors

## Abstract

**Background:**

This study aims to evaluate inequality in oral health among adolescents and to explain the mechanisms of such inequalities in Gangneung, South Korea.

**Methods:**

One thousand two hundred sixty-seven students in their first year from four vocational and three general schools participated in the baseline survey of 2011, and 84.7% of them were surveyed again in 2013. Oral examinations by the same dentist and a self-administered questionnaire were repeated during both waves. Outcome measure for oral health was the existence of untreated dental caries (DT). As socioeconomic position (SEP) indicators, school type (general vs. vocational), father’s and mother’s education, perceived economic status, and Family Affluence Scale (FAS) were measured. Variables measuring oral health related behaviours included tooth brushing frequency, frequency of eating snacks and drinking sodas, smoking, and annual visits to dental clinics. Chi-square tests and panel logistic regression were adopted to examine the associations between dental caries and SEP indicators by STATA version 15.1.

**Results:**

Having a less educated father and attending a vocational school were significant predictors for untreated caries after controlling for SEP indicators. However, students from general schools, higher SEP by father’s education, perceived economic status, or FAS, or having non-smoking experience or annual visits to dental clinics were more likely to stay caries-free.

**Conclusions:**

There were socioeconomic inequalities in oral health on an adolescent panel. Given that oral health status during adolescents can persist throughout the course of a person’s life, intervention to tackle such inequalities and school environments are required.

**Electronic supplementary material:**

The online version of this article (10.1186/s12903-018-0533-3) contains supplementary material, which is available to authorized users.

## Background

Various studies have recognized that there are socioeconomic inequalities in health [1–3] and oral health [4, 5]. A gap exists across all levels of socioeconomic groups, especially between the highest and the lowest ones. Furthermore, such inequalities are observed throughout the course of a person’s life, from childhood to adulthood and into old age [6–9]. However, there are differing opinions for the existence of health inequality in a specific life stage: adolescence.

West [10, 11] suggested that adolescents exhibit dissimilar health inequality patterns when compared to other age groups because this group can be “characterised by the absence or disappearance of class variation.” To the contrary, others have claimed that findings on health equality among adolescents are the result of inappropriate measurements for socioeconomic position (SEP) indicators [12, 13]. These studies argued that commonly used SEP indicators, including education, occupation, and income levels, are not appropriate for adolescents. For example, there was a significant health gap in adolescents when using alternative SEP indicators such as the Family Affluence Scale (FAS) [14] and perceived socioeconomic status [15]. Subsequent to these debates, studies in different settings have attempted to verify both positions but a consensus stills has not been reached [16–18].

Recently, there have been several longitudinal studies dealing with inequality in adolescent oral health by SEP indicators. A life-course research in cohort of New Zealand children concluded that there was an effect from childhood SEP [19]. A study from Sweden showed that there is limited effect from SEP only after considering the previous experience of caries [20]. Polk et al. [21] and Newacheck [22] also reported that there was an SEP gradient in caries experiences in the U.S. Another longitudinal study from Iowa cohort revealed that there is a gap by SEP, especially maternal educational levels [23]. However, Curtis, using the same cohort data from Iowa Fluoride Study (IFS), recently argued that ‘the role of SES in caries may not be as important as previously thought’. Meanwhile, another group of scholars have focused on school characteristics as an alternative to traditional SEP indicators [24–27] or influential effectors for behaviour, especially in S. Korea [28, 29], to determine health inequalities in adolescents. Others have reported that the residential area was a socioeconomic predictor of oral health inequality among adolescents [30, 31].

In terms of oral health, adolescence is of special importance because permanent dentition is complete and parental supervision of oral health behaviours weakens. Indeed, dental caries is a unique health condition aggravating during the schooling period. On the other hand, as they are amenable by health education and highly receptive to public health programs, interventions may be effective and the effects may be long lasting.

This study aims to evaluate inequality in oral health among adolescents by various SEP indicators and to explain the mechanisms of such inequalities in Gangneung, a city in South Korea.

## Methods

### Study participants

The aim of this panel study was to explore inequality in untreated caries among adolescents. The city of Gangneung has eleven high schools; two in rural areas and nine in urban areas [32]. By the school types, there were six general schools, four vocational schools, and one art school. The aim of the general schools is to support students’ academic development and entry into college. By contrast, the aim of the vocational schools is to prepare students to enter the workforce directly after graduation. The one art school in this study was excluded due to its unique characteristics. First, the schools were categorized by its location as an urban or rural area. There was only one general and one vocational school in the rural area, so both of them were included. Among the other eight schools in the urban area, three vocational schools and two general schools were selected as study samples. Because they have fewer students than the general schools, all of the vocational schools were included. Two general schools were randomly selected by their close proximity to sampled vocational schools. Only first year students, 15-yr-old, were invited to participate in consideration of the follow-up survey after two years. All of the students were sixteen years old because middle school education is mandatory in South Korea, so the freshman in high school were all the same age. The research team sent the consent forms for oral examinations and surveys to students’ parents or guardians with a brief introduction. Only students who returned completed consent forms from their parents or guardians were included in the study. Among 1371 students, ninety-seven declined to participate, and seven were excluded due to incomplete answers on the questionnaires. Finally, 1267 students were enrolled into the panel.

The Institutional Review Board in Gangneung-Wonju National University Dental Hospital reviewed and approved this study (GWNUDH IRB-2011-1-3). The Gangneung Health Centre and the Gangneung Office of Education with their district offices also consented to the study and supported the administrative process.

### Study variables and measurement

Outcome measure for oral health was the untreated dental caries according to the Korean National Oral Health Survey (KNOHS) standard [33], which follows the guideline established by the WHO methods for oral health survey [34]. The untreated dental caries “D” component, which includes carious teeth, filled teeth with recurrent decay, teeth with only the root left, defective filling with caries, temporary filling, and teeth with a filled tooth surfaces but with other surface decayed.

The examinations were conducted in a classroom of the surveyed schools using their table and chairs with a lightweight portable examination light. The plane mouth mirrors, periodontal probes that conform to WHO specifications, and several pairs of tweezers were supplied for the survey. The same dentist who was trained according to the Korean National Oral Health Survey (KNOHS) confirmed the students’ oral status twice in three years. To have reliable intra-examiner reliability, the dentist examined 20 students before the main study. He re-examined them one week later for calibration, and the kappa consistency was 0.91, good agreement [35]. The status transition in dental caries between 2011 and 2013 were classified as follows: 1) no to no (remained caries-free); 2) yes to no (received treatment); 3) no to yes (developed new dental caries); and 4) yes to yes (remained untreated caries).

Self-administered questionnaire surveys were administered, and items were derived from the Korean Youth Risk Behaviour Web-based Survey (KYRBWS) [36, 37] and from the guidelines in “Delivering better oral health: an evidence-based toolkit for prevention” [38]. As SEP indicators, school type (general vs. vocational), father’s and mother’s education level, perceived economic status, and FAS were measured. Father’s and mother’s education level was categorized into two groups; high school graduation or below vs. college graduation or above. The perceived economic status were re-categorised as high (high, high-middle, and middle) vs. low (middle-low and low). The FAS score was calculated by summing up dummy variables to represent the ownership of a family car, private bedroom for the student, computers, and the number of family vacations in the past year. FAS scores range from zero to nine, and students with scores larger than five were defined as ‘high’ and the others as ‘low’ group. Variable to measure oral health related behaviours included tooth brushing frequency (‘twice or more a day’ vs. ‘less than twice a day’), frequency of snacks and drinking sodas (‘less than once a day’ vs. ‘once or more a day’), smoking (‘no’ vs. ‘yes’), and annual visits of dental clinics at least once a year (‘yes’ or ‘no’).

### Statistical analysis

Annual prevalence of untreated caries (decayed teeth; ‘D rate’) and proportion of status transition in dental caries over the follow-up was examined according to various SEP indicators and oral health related behaviours by chi-square tests. In order to identify independent effects of SEP variables and contributions of covariates to oral health status with considering panel design, unconditional panel logistic regression models were estimated. Data analysis was carried out using STATA version 15.1 statistical software package (StataCorp, Texas).

## Results

The characteristics of the study participants are displayed in the Table 1. At the baseline survey of 2011, the participation rate was 92.4%, and a total of 1267 students participated. The follow-up rate was 84.7% in 2013 with the drop of 194 students. Attrition was more common in vocational schools (27.3%) than general schools (7.4%). However, this did not present significant changes in the distribution of gender and SEP indicators of the sample between waves (Additional file 1: Table S1).

**Table 1:** The characteristics of study participants by school type in Gangneung

School location	School type	Sampled population *N*	Respondents in baseline 2011 (1st grade, 15-yr-old) *N*	Gender		Respondents in follow-up 2013 (3rd grade, 17-yr-old) *N*
				Boys *N (%)*	Girls *N (%)*	
Total		1371	1267	690(54.5)	577(45.5)	1073
Rural						
School A	General	93	88	41(46.6)	47(53.4)	71
School B	Vocational	155	133	48(36.1)	85(63.9)	99
Urban						
School C	General	341	335	335(100.0)		314
School D	General	360	343		343(100.0)	324
School E	Vocational	296	242	242(100.0)		168
School F	Vocational	58	58	24(41.4)	34(58.6)	45
School G	Vocational	68	68		68(100.0)	52

At both waves, the students who were from vocational schools, less educated fathers, and ‘low’ groups of perceived economic status and FAS were more likely to have untreated dental caries. As for oral health behaviours, tooth brushing and annual visits were inversely associated with D rates. Smoking was strongly associated with D rates in both waves (Table 2).

**Table 2:** Numbers and percentages of adolescents who have untreated dental caries (D rate) by survey year in Gangneung

D rate	201l (1st grade, 15-yr-old) *N* = 1267		2013 (3rd grade, 17-yr-old) *N* = 1073	
Total	217(17.1)		153(14.3)	
Gender				
Girls	92(15.9)		62(12.2)	
Boys	125(18.1)		91(16.1)	
School type				
General	94(12.3)	***	70(9.9)	***
Vocational	123(24.6)		83(22.8)	
Father’s education				
College or above	50(10.5)	***	33(7.7)	***
High school or below	137(19.8)		98(17.0)	
Mother’s education				
College or above	46(13.3)		31(10.3)	
High school or below	143(17.6)		101(14.4)	
Perceived economic status				
High	145(15.6)	*	99(12.6)	**
Low	72(21.4)		54(19.2)	
FAS				
High	171(15.6)	***	115(12.5)	***
Low	46(27.4)		38(26.0)	
Frequency of tooth-brushing				
≥2	182(16.2)	*	137(14.0)	
< 2	35(24.5)		16(17.4)	
Frequency of eating snacks				
< 1	71(15.7)		118(13.4)	*
≥1	131(17.3)		35(18.9)	
Frequency of drinking soda				
< 1	99(15.1)		134(13.6)	
≥1	95(18.6)		19(21.1)	
Smoking experience				
No	124(13.8)	***	91(12.0)	**
Yes	93(25.3)		62(19.6)	
Annual visits to dental clinic				
Yes	84(14.6)	*	55(10.7)	**
No	133(19.2)		98(17.6)	

*p < 0.05, **p < 0.01, ***p < 0.001

The odds ratios (ORs) for untreated caries were estimated after adjusting for SEP indicators only in Model 1, and SEP indicators and oral health related behaviours covariates together in Model 2 (Table 3). As for D rates, fathers’ education and school type remained significant after controlling for other SEP indicators. Even after incorporating health behaviour variables in the models, they still showed significant effects with attenuation.

**Table 3:** Adjusted odds ratio and 95% confidence intervals estimated from unconditional panel logistic regression models for D rate among adolescents in Gangneung (*N* = 2003)

D rate	Model 1†		Model 2‡	
Gender				
Girls (vs. boys)	0.65(0.39–1.06)		0.71(0.41–1.23)	
School type				
Vocational (vs. general)	3.10(1.79–5.35)	***	2.68(1.46–4.92)	**
Father’s education				
High school (vs. college)	2.15(1.15–4.01)	*	2.04(1.05–3.98)	*
Mother’s education				
High school (vs. college)	0.92(0.48–1.76)		0.86(0.43–1.73)	
Perceived economic status				
Low (vs. high)	1.03(0.62–1.71)		1.05(0.61–1.81)	
FAS				
Low (vs. high)	1.82(0.95–3.49)		1.80(0.88–3.64)	
Frequency of tooth-brushing				
< 2 (vs. N ≥ 2)			1.42(0.72–2.83)	
Frequency of eating snacks				
≥1 (vs. *N* < 1)			1.64(1.00–2.70)	
Frequency of drinking soda				
≥1 (vs. N < 1)			1.13(0.66–1.95)	
Smoking experience				
Yes (vs. no)			1.72(1.02–2.92)	*
Annual visits to dental clinic				
No (vs. yes)			1.91(1.22–3.01)	**

† Model 1: adjusted for gender, school type, father’s education, mother’s education, subjective economic status, and FAS

‡ Model 2: adjusted for gender, school type, father’s education, mother’s education, subjective economic status, FAS, frequency of tooth brushing, eating snacks, and drinking soda, smoking, and annual visits to dental clinics

*p < 0.05, **p < 0.01, ***p < 0.001. - mean VIF (=1.37) < 10

The status transition in dental caries over the follow-up is shown in Table 4. Students from general schools, in higher SEP measured by father’s education, perceived economic status, or FAS, drinking soda less than a day, without smoking experience, and to have annual visits to dental clinics were more likely to stay caries-free (*p* < 0.01).

**Table 4:** Condition transition for untreated caries over the two-year follow-up in Gangneung adolescent at 2013 (*N* = 1073)

	Caries status change (from 2011 to 2013)				
	No → No^a^	Yes → No^b^	No → Yes^c^	Yes → Yes^d^	
Total	834 (77.7)	86 (8.1)	61 (5.7)	92 (8.6)	
Gender					
Girls	397 (78.3)	48 (9.5)	25 (4.9)	37 (7.3)	
Boys	437 (77.2)	38 (6.7)	36 (6.4)	55 (9.7)	
School type					***
General	585 (82.5)	54 (7.6)	37 (5.2)	33 (4.7)	
Vocational	249 (68.4)	32 (8.8)	24 (6.6)	59 (16.2)	
Father’s education					***
College or above	361 (84.5)	33 (7.7)	23 (5.4)	10 (2.3)	
High school or below	432 (74.9)	47 (8.2)	34 (5.9)	64 (11.1)	
Mother’s education					
College or above	244 (81.1)	26 (8.6)	18 (6.0)	13 (4.3)	
High school or below	545 (77.8)	55 (7.9)	38 (5.4)	63 (9.0)	
Perceived economic status					**
High	621 (78.8)	68 (8.6)	47 (6.0)	52 (6.6)	
Low	209 (74.4)	18 (6.4)	14 (5.0)	40 (14.2)	
FAS					***
High	732 (79.2)	77 (8.3)	52 (5.6)	63 (6.8)	
Low	100 (68.5)	8 (5.5)	9 (6.6)	29 (19.9)	
Frequency of tooth-brushing					
≥2	764 (77.9)	80 (8.2)	53 (5.4)	84 (8.6)	
< 2	70 (76.1)	6 (6.5)	8 (8.7)	8 (8.7)	
Frequency of eating snacks					
< 1	699 (79.1)	67 (7.6)	47 (5.3)	71 (8.0)	
≥1	131 (70.8)	19 (10.3)	14 (7.6)	21 (11.4)	
Frequency of drinking soda					**
< 1	777 (79.0)	72 (7.3)	53 (5.4)	81 (8.2)	
≥1	57 (63.3)	14 (15.6)	8 (8.9)	11 (12.2)	
Smoking experience					***
No	611 (80.8)	54 (7.1)	42 (5.6)	49 (6.5)	
Yes	223 (70.4)	32 (10.1)	19 (6.0)	43 (13.6)	
Annual visits to dental clinic					**
Yes	410 (79.5)	51 (9.9)	18 (3.5)	37 (7.2)	
No	424 (76.1)	35 (6.3)	43 (7.7)	55 (9.9)	

^a^No → No as remaining caries-free, ^b^Yes → No as being treated with fillings, ^c^No → Yes as developing dental caries, and ^d^Yes → Yes as remaining untreated caries

**p < 0.01, ***p < 0.001

## Discussion

Our analysis of the adolescent panel in Gangneung, South Korea, verified the existence of significant differences in untreated dental caries by school type and father’s education, and in caries experience by gender and father’s education. Oral health-related behaviours attenuated but did not explain away such effects.

Differences in oral health by school type can be attributed to the fact that schools are a place where youths form a unique culture. Here, they begin to be independent from their family, spending most of the day with their peers. In a society where college graduation is the norm, vocational schools could be a symbolic representation of social disadvantages, which are related to poor health and undesirable health behaviours. There was a report that male and female students in Korean vocational schools are more likely to engage in non-conformant behaviours, including smoking, alcohol consumption, unexcused absences, running away from home, and sexual relationships, as well as to be involved in assault, harassment, and adolescent prostitution [39]. Another Korean study found that even after controlling for individual-level SEP indicators and psychological stress, students in vocational schools engaged more in risky behaviours, for example smoking and drinking, and less in health-promoting behaviours, such as tooth brushing [40, 41].

There was no significant difference by gender in untreated dental caries. It is contrary to the findings in the 2012 Korean National Oral Health Survey, which showed higher prevalence of decayed teeth among girls by 10.6% [42]. Martinez-Mier and Zandona [43] argued that caries is multifactorial disease attributable to diverse factors, including genetic and hormonal factors as well as cultural influences, behavioural, and dietary practices. Kawamura and colleagues attributed the better oral health of girls to more frequent tooth brushing and their desires to possess healthy teeth [44]. It was suggested that their concern for good oral health motivates them to visit dental clinics for treatment more often than males. Indeed, we found that students who visited dental clinics more regularly had a reduced likelihood of having untreated caries; adolescents who visited the dental clinic more regularly to receive treatment had less untreated caries.

It is noteworthy that father’s education but not mother’s education was a strong predictor of oral health in Korean adolescents. This is contrary to the report that mothers play an important role in the child development of oral health [45]. First, the cut-off level of mother’s education might be inappropriate to capture the differences; in fact, only 30% of students in the panel had mothers who had graduated college, which means that the ‘low’ education group consisted of heterogeneous individuals, resulting in non-differential misclassification. Second, although parents’ education level is associated with health literacy and awareness and thereby children’s behaviours and service utilisation [46], parental influence wanes during transitional developmental periods [47]. Children have a desire to be similar with their friends, and such desires become stronger in older adolescents [48]. Therefore, mother’s education might have little impact on the oral health and behaviours of adolescents in this panel. Along the same line, in our study, father’s education as surrogate marker of household material conditions might be a better indicator of cultural and behavioural resources than mother’s education. In Korea, fathers are generally family breadwinners, and there is a clear correlation between fathers’ education and family economic status. As a mid-sized semi-urban city, Gangneung provides less job opportunities. In this context, father’s education could surpass other SEP indicators in representing household socioeconomic conditions.

The oral health behaviours of the study panel improved during the follow-up. For instance, the prevalence of eating less snacks went from under 37.5% at baseline to 82.7% at the second wave. Students exhibiting healthier behaviours increased dramatically in terms of oral health; they brushed teeth more and consumed less snacks and soda. All of this generally contributes to better oral health [49–51], and indeed D rates with untreated caries in our panel declined over time.

This study has some limitations. First, although differential sample attrition between general and vocational schools did not bring out differences in the distribution of socioeconomic factors among students, we cannot be sure that there was no systematic difference in oral health-related behaviours between participants and dropouts. Second, we could not fully explain why the strong effect of father’s education persisted even after controlling for oral health-related behaviours. Third, because the panel was composed of high-school students only, it was hard to conduct follow-ups after their graduation. In order to examine long-term effects of oral health and related behaviours in adolescents, it would thus be necessary to expand the panel range to primary school students as they begin to formulate health behaviours.

## Conclusion

We found socioeconomic inequalities in oral health based on an adolescent panel from Gangneung. Given that poor oral health and undesirable oral health-related behaviours during the adolescent period could last throughout a person’s lifetime [52], there should be an immediate intervention to tackle such inequalities and school environments.

## Additional file

Additional file 1: **Table S1.** The characteristics of the participants by sociodemographic information in Gangneung. (DOCX 14 kb)
