# Supplementary material for: Inequalities in oral health among adolescents in Gangneung, South Korea
Source: BMC Oral Health. 2018 Apr 24;18:68. doi: 10.1186/s12903-018-0533-3 (PMC5921975; doi:10.1186/s12903-018-0533-3)
Supplement: Supplementary file 1 — Table S1. The characteristics of the participants by sociodemographic information in Gangneung. (DOCX 14 kb) [file 12903_2018_533_MOESM1_ESM.docx]

Table S1: The characteristics of the participants by sociodemographic information in Gangneung

| Participants | 201l  (1^st^ grade,  15-yr-old) | | 2013  (3^rd^ grade,  17-yr-old) | |
| --- | --- | --- | --- | --- |
| Total | 1,267(100.0) |  | 1,073(100.0) |  |
| Gender |  |  |  |  |
| Girls | 690(54.5) |  | 566(52.8) |  |
| Boys | 577(45.5) |  | 507(42.3) |  |
| School type |  |  |  |  |
| General | 766(60.5) |  | 709(66.1) | ^**^ |
| Vocational | 501(39.5) |  | 364(33.9) |  |
| Father’s education |  |  |  |  |
| College or above | 476(40.8) |  | 427(42.5) |  |
| High school or below | 691(59.2) |  | 577(57.5) |  |
| Mother’s education |  |  |  |  |
| College or above | 346(29.8) |  | 301(30.0) |  |
| High school or below | 814(70.2) |  | 701(70.0) |  |
| Perceived economic status |  |  |  |  |
| High | 930(73.4) |  | 788(73.7) |  |
| Low | 337(26.6) |  | 281(26.3) |  |
| FAS |  |  |  |  |
| High | 1,099(86.7) |  | 924(86.4) |  |
| Low | 168(13.3) |  | 146(13.6) |  |

^*^p < 0.05, ^**^ p < 0.01, ^***^p < 0.001.
